# Pure Fruit Juice and Fruit Consumption Are Not Associated with Incidence of Type 2 Diabetes after Adjustment for Overall Dietary Quality in the European Prospective Investigation into Cancer and Nutrition–Netherlands (EPIC-NL) Study

**DOI:** 10.1093/jn/nxz340

**Published:** 2020-01-14

**Authors:** Floor R Scheffers, Alet H Wijga, W M Monique Verschuren, Yvonne T van der Schouw, Ivonne Sluijs, Henriëtte A Smit, Jolanda M A Boer

**Affiliations:** 1 Center for Nutrition, Prevention, and Health Services, National Institute for Public Health and the Environment (RIVM), Bilthoven, Netherlands; 2 Julius Center for Health Sciences and Primary Care, University Medical Center Utrecht, Utrecht University, Netherlands; 3 Faculty of Health, Nutrition, and Sport, The Hague University of Applied Sciences, The Hague, Netherlands

**Keywords:** pure fruit juice, fruit, diabetes mellitus type 2 incidence, dietary guidelines, prospective cohort study, EPIC-NL

## Abstract

**Background:**

Dietary guidelines on pure fruit juice consumption vary from country to country regarding the inclusion of pure fruit juice in the recommendations as an acceptable alternative for fruit. Current epidemiological evidence on the association between pure fruit juice consumption and diabetes risk is scarce.

**Objective:**

We studied the association of both pure fruit juice and fruit consumption with diabetes risk and investigated the differences between low and high fruit consumers in the association of pure fruit juice consumption with diabetes risk.

**Methods:**

This prospective cohort study included 36,147 participants in the European Prospective Investigation into Cancer and Nutrition–Netherlands (EPIC-NL) Study aged 20–69 y at baseline. Fruit juice and fruit consumption were assessed using a validated food-frequency questionnaire; amounts of consumption were divided into 5 categories and quintiles, respectively. Incident type 2 diabetes cases were mainly self-reported and verified against medical records. Cox regression was used to estimate adjusted HRs and 95% CIs.

**Results:**

After an average follow-up of 14.6 y, 1477 verified incident cases of type 2 diabetes were documented. Compared with no consumption, pure fruit juice consumption was not significantly associated with type 2 diabetes, with adjusted HRs ranging from 0.92 (95% CI: 0.79, 1.09) to 1.03 (95% CI: 0.83, 1.26). The associations did not differ between participants with low and high fruit consumption. None of the categories of fruit consumption were associated with type 2 diabetes (lowest quintile as reference). Adjusted HRs ranged between 0.93 (95% CI: 0.78, 1.10) and 1.00 (95% CI: 0.84, 1.19). Adjustment for the Dutch Healthy Diet Index, as an overall measure of dietary quality, strongly attenuated the observed associations of type 2 diabetes with both fruit juice and fruit consumption.

**Conclusions:**

We found no evidence for associations between pure fruit juice and fruit consumption and diabetes risk after adjustment for overall dietary quality for participants in the EPIC-NL study. This trial was registered at https://www.trialregister.nl/trial/6939 as NL6939.

## Introduction

Recent meta-analyses of epidemiological studies on the association between fruit consumption and type 2 diabetes showed that fruit consumption is associated with a lower risk of type 2 diabetes (1–3). Less is known about the association between pure fruit juice consumption and type 2 diabetes. Pure fruit juice can be both freshly squeezed and bottled juice and never contains added sugars, artificial sweeteners, flavorings, preservatives, or colorings. It therefore differs from sugar-sweetened beverages (SSBs). Dietary guidelines on pure fruit juice consumption vary from country to country (4). In the United States, dietary guidelines state that pure fruit juice can count for half of the recommended fruit intake per day (5). In the United Kingdom, pure fruit juice should not count for >1 serving/d and should be restricted to a portion size of 150 mL (6). A few countries (e.g., The Netherlands) classify pure fruit juice in the category “sugar-containing beverages” because of the sugar content that is comparable to that of sugar-sweetened soft drinks and state that consumption should be restricted to a minimum (7). Several epidemiological studies showed that the consumption of SSBs—which make up a large proportion of all sugar-containing beverages—is associated with a higher risk of developing type 2 diabetes (8–12). These conflicting dietary guidelines underscore that more knowledge is needed on the health effects of pure fruit juice consumption. However, epidemiological evidence on the association between pure fruit juice and the risk of type 2 diabetes is scarce and inconsistent (8–11, 13). Therefore, we investigated the association of pure fruit juice consumption with the incidence of type 2 diabetes in the European Prospective Investigation into Cancer and Nutrition (EPIC)–Netherlands (EPIC-NL) cohort. Furthermore, we examined whether pure fruit juice consumption is differentially associated with the risk of type 2 diabetes in low versus high fruit consumers. This is because the guidelines from the United States and United Kingdom state that part of the fruit consumption may be replaced by pure fruit juice consumption (5, 6). As a secondary objective, we also studied the association of fruit consumption with the incidence of type 2 diabetes.

## Methods

### Study population

The EPIC-NL study consists of the 2 Dutch cohorts (Prospect and MORGEN) that contribute to the EPIC. For both cohorts, participants were recruited between 1993 and 1997. The MORGEN-EPIC cohort includes 22,654 men and women aged 20–65 y at baseline, who were selected from random samples of 3 Dutch towns in the Netherlands (Amsterdam, Doetinchem, and Maastricht). The Prospect-EPIC cohort includes 17,357 women aged 49–70 y who were participating in the national breast-cancer-screening program and living in the Dutch city of Utrecht or its surroundings. The design of the EPIC-NL study has been described elsewhere (14). All participants provided written informed consent before they were included in the study. The study complies with the Declaration of Helsinki and was approved by the Institutional Review Board of the University Medical Center Utrecht (Prospect) and the Medical Ethical Committee of TNO Nutrition and Food Research (MORGEN). The present study was registered as https://www.trialregister.nl/trial/6939 as NL6939.

### Exposure assessment

A self-administered food-frequency questionnaire (FFQ) was used to assess daily nutritional intake. This FFQ contained questions on the usual frequency of consumption of 178 food items during the year preceding enrollment. The FFQ has been validated against the mean of twelve 24-h recalls. For fruit consumption, the relative validities of ranking of subjects using the FFQ with respect to using the mean of twelve 24-h recalls expressed as Spearman's correlation coefficients between the 2 were 0.68 in men and 0.56 in women (15). The FFQ contained 2 questions on pure fruit juice consumption. The first question was, “How many glasses of fruit juice do you habitually drink?” Participants indicated their consumption in glasses per day, per week, per month, per year, or as never. The second question was, “What kind of fruit juices do you habitually drink?” The prespecified choices were apple juice, orange/grape fruit juice, and other fruit juice. Fruit consumption in winter and fruit consumption in summer were assessed with separate questions and therefore the FFQ contained 4 questions on fruit consumption. These questions were as follows: “How many pieces/portions of fruit do you habitually consume in summer?” and “How many pieces/portions of fruit do you habitually consume in winter?” Subsequently, participants were asked “Which kinds of fruit do you eat in summer?” and “Which kinds of fruit do you eat in winter?” The prespecified choices for summer fruits were apple/pear, citrus fruit, banana, strawberry, grapes, peach, cherries, kiwi, melon, and “other, namely…,” whereas the prespecified choices for winter fruits were apple/pear, citrus fruit, banana, kiwi, and “other, namely…”.

### Outcome assessment

A 2-step approach was used for the identification and verification of potential type 2 diabetes cases. For the identification of potential cases, information was obtained through linkage with the hospital discharge diagnosis registry and from follow-up questionnaires. In the hospital discharge diagnoses registry, all diagnoses were coded according to the *International Classification of Diseases, Ninth Revision, Clinical Modification* (ICD-9-CM). Potential cases were identified based on code 250 and underlying codes. The follow-up questionnaires included a question on whether a diagnosis of diabetes had been made and were sent out at intervals of 3 to 5 y (years 1998–2002, questionnaire 1; 2003–2007, questionnaire 2; and 2011–2012, questionnaire 3). Prospect participants additionally received a urinary glucose strip test with the first questionnaire. They were asked to self-report whether the strip had turned purple after 10 s, indicating glucosuria. All potential type 2 diabetes cases up to 2006 were validated by consulting the general practitioner or the pharmacist (16). For all potential cases identified after 2006, only the general practitioner was used as the verification source. The verification source provided the year of diagnosis; for purposes of analysis, we set the diagnosis date for all identified cases at 1 January of the year of diagnosis. Follow-up ended at 31 December 2010.

### Covariates

Educational level was coded as low (primary education, lower vocational education, advanced elementary education), intermediate (intermediate vocational education, completion of first 3 y of higher general secondary education), or high (completed higher general secondary education, higher vocational education, and university). Family history of diabetes was classified as none, 1 parent, both parents or unknown. Smoking (cigarette, cigar, or pipe) was categorized into current, former, or never. Alcohol consumption was categorized into 4 categories (<10 g ethanol/d, 10 to <20 g ethanol/d, 20 to <30 gram ethanol/d, or ≥30 g ethanol/d). Physical activity was assessed using a questionnaire validated in an elderly population (17) and classified according to the Cambridge Physical Activity Index as a dichotomous variable (moderately active/active and moderately inactive/inactive) (18). We were not able to calculate a total physical activity score for 14% of all participants. Therefore, we imputed these missing scores using single imputation (SPSS MVA procedure) (19). The Dutch Healthy Diet Index 2015 (DHD15-index), based on the adherence to the Dutch dietary guidelines of 2015 (7), was used as a measure of diet quality. The DHD15-index consists of 15 components: vegetables, fruit, whole-grain products, legumes, nuts, dairy, fish, tea, fats and oils, filtered coffee, red meat, processed meat, sugar-sweetened beverages, alcohol, and salt. For each component, a score between 0 (no adherence) and 10 (complete adherence) was calculated. More detailed information on the calculation of the DHD15-index has been published elsewhere (20). In the EPIC-FFQ, no information was available on the type of coffee consumed (filtered or unfiltered). Therefore, the component “coffee” was excluded from the DHD15-index we used. Since fruit was the exposure variable of interest in our study, this item was also excluded. Furthermore, we excluded sugar-sweetened beverages from the DHD15-index and adjusted for this covariate separately. Consequently, our DHD15-index consisted of 12 components and ranged from 0 (no adherence) to 120 (complete adherence). BMI was calculated by dividing weight by height squared (kg/m^2^).

### Statistical analyses

Characteristics of the study population were summarized using descriptive statistics. Cox proportional hazards regression was performed to determine HRs and 95% CIs for the association of pure fruit juice consumption and fruit consumption with incidence of type 2 diabetes.

Pooled HRs were estimated using stratified Cox models assuming different baseline hazards for the 2 cohorts. Fulfillment of the proportional hazard assumption was tested with Schoenfeld residuals. For pure fruit juice, we used a standard portion size of 150 mL/glass. We converted the total consumption of pure fruit juice into glasses of 150 mL/wk and divided this into 5 categories; nondrinkers, <1 glass/wk, 1 to <4 glasses/wk, 4 to <8 glasses/wk, or ≥8 glasses/wk (nondrinkers as reference). Associations with fruit consumption were analyzed based on quintiles with the lowest quintile used as reference. Selection of potential confounders was based on a priori theoretical considerations derived from the scientific literature. A first model was adjusted for age and sex. A second model was adjusted for age, sex, educational level, physical activity, smoking, family history of diabetes, alcohol consumption, coffee consumption, DHD15-index, and fruit consumption (for associations with pure fruit juice consumption) or pure fruit juice consumption (for associations with fruit consumption). Additionally, in a third model energy intake and in a fourth model BMI and waist circumference (but not energy intake) were added as covariates to elucidate the role of these potential intermediate factors in the association between pure fruit juice/fruit consumption and incidence of type 2 diabetes. We present associations of pure fruit juice with incidence of type 2 diabetes incidence separately for participants with low fruit consumption and those with high fruit consumption in order to investigate possible benefits of pure fruit juice consumption for low fruit consumers (lowest 2 quintiles: ≤120 g/d) compared with high fruit consumers (highest 3 quintiles: >120 g/d). The hypothesized difference in association of pure fruit juice consumption between participants with a low and those with a high fruit consumption was tested by including interaction terms using a significance level of *P* < 0.10. We also included interactions terms to test possible effect modification by sex, physical activity, and BMI. *P* values for linear trend across the categories of pure fruit juice consumption and quintiles of fruit consumption were calculated by including fruit and pure fruit juice consumption as continuous variables in the models. All analyses were performed using SAS, version 9.4 (SAS Institute, Inc.).

For the present study, participants who withdrew permission for inclusion in the study were excluded (*n* = 1). Additionally, we excluded participants with no information on dietary intake (*n* = 218); those with extremely low or high reported energy intake (i.e., those in the lowest or highest 0.5% of the ratio of energy intake over basal metabolic rate) (*n* = 390); those missing follow-up (*n* = 1738); those with prevalent diabetes at baseline, nonverified incident diabetes, and unknown type of incident diabetes (*n* = 1241); or those missing data on any of the covariates (*n* = 276). In total, 36,147 participants were included in the analyses (**Supplemental Figure 1**).

### Sensitivity analyses

Verification information was available for 1477 potential type 2 diabetes cases but was not available for 490 potential diabetes cases, mainly because the general practitioner could not be traced or did not respond. Sensitivity analyses were performed including these participants as diabetes cases.

Since orange/grapefruit and apple juices were mostly pure fruit juices at the time of assessment, we assumed that reporting not 100% fruit juice as pure fruit juice is unlikely for these types. However, we assumed that “other fruit juice” is more vulnerable for misreporting and therefore we conducted a sensitivity analysis in which we excluded this subitem. Furthermore, we analyzed the association with incidence of type 2 diabetes separately for the consumption of orange/grapefruit and apple juice to explore possible differences between these types of pure fruit juices.

## Results

During a mean ± SD follow-up of 14.6 ± 3.0 y, 1477 verified incident cases of type 2 diabetes were documented. **Table 1** shows the distribution of the baseline characteristics of the 36,147 participants over the categories of fruit juice consumption; 25.5% were men and 74.5% were women and the mean ± SD age was 49 ± 11.9 y. Participants had a median pure fruit juice consumption of 40 g/d (IQR: 119 g) and a median fruit consumption of 128 g/d (IQR: 160 g). Of participants, 15.7% reported that they did not consume pure fruit juice and 1.0% reported that they did not consume fruit at all. Compared with nonconsumers, pure fruit juice drinkers were more often women and nonsmokers. They had a higher educational level, were more physically active, and were less often heavy alcohol consumers. They also had healthier dietary habits. We found similar results for participants with a high fruit consumption compared with participants with a low fruit consumption, with the exception of age: high fruit consumers were older than low fruit consumers, whereas pure fruit juice drinkers were younger than nondrinkers (Table 1 and **Supplemental Table 1**).

**TABLE 1:** Baseline characteristics according to categories of pure fruit juice consumption^1^

		Categories of pure fruit juice consumption^2^				
	All participants	Nondrinkers (*n* = 5669)	<1 glass/wk (*n* = 7874)	1 to <4 glasses/wk (*n* = 9782)	4 to <8 glasses/wk (*n* = 9561)	≥8 glasses/wk (*n* = 3261)
Cohort						
Prospect	43.5 (15,716)	47.7 (2706)	40.3 (3172)	40.3 (3939)	48.8 (4667)	37.8 (1232)
MORGEN	56.5 (20,431)	52.3 (2963)	59.7 (4702)	59.7 (5843)	51.2 (4894)	62.2 (2029)
Sex						
Male	25.5 (9224)	29.2 (1656)	29.1 (2290)	26.8 (2623)	19.3 (1848)	24.8 (807)
Educational level^3^						
Low	57.6 (20,810)	73.7 (4179)	54.7 (4303)	53.5 (5235)	55.4 (5301)	55.0 (1792)
Intermediate	21.9 (7927)	15.1 (857)	23.6 (1856)	23.5 (2296)	22.4 (2145)	23.7 (773)
High	20.5 (7410)	11.2 (633)	21.8 (1715)	23.0 (2251)	22.1 (2115)	21.3 (696)
Family history of diabetes						
None	76.7 (27,725)	74.1 (4199)	76.8 (6049)	77.8 (7610)	77.1 (7369)	76.6 (2498)
One parent	17.0 (6156)	18.7 (1057)	16.7 (1314)	16.4 (1599)	17.1 (1631)	17.0 (555)
Both parents	0.9 (326)	0.9 (50)	1.0 (80)	0.9 (87)	0.9 (85)	0.7 (24)
Unknown	5.4 (1940)	6.4 (363)	5.5 (431)	5.0 (486)	5.0 (476)	21.3 (696)
Smoking status						
Never	38.2 (13,792)	28.6 (1621)	36.3 (2857)	39.9 (3898)	42.0 (4016)	42.9 (1400)
Former	31.4 (11,338)	32.5 (1842)	33.3 (2620)	31.0 (3032)	30.7 (2934)	27.9 (910)
Current	30.5 (11,017)	38.9 (2206)	30.4 (2397)	29.2 (2852)	27.3 (2611)	29.2 (951)
Physical activity^4^						
Inactive to moderately inactive	32.0 (11,556)	37.9 (2151)	32.0 (2516)	30.6 (2988)	30.9 (2957)	29.0 (944)
Moderately active to active	68.0 (24,591)	62.1 (3518)	68.1 (5358)	69.5 (6794)	69.1 (6604)	71.1 (2317)
Alcohol intake						
Never	0.5 (165)	1.4 (81)	0.2 (17)	0.2 (19)	0.4 (33)	0.5 (15)
<10 ethanol, g/d	62.7 (22,658)	59.6 (3381)	62.2 (4896)	62.5 (6110)	62.9 (6016)	69.2 (2255)
10 to <20 ethanol, g/d	16.5 (5954)	15.0 (849)	17.0 (1339)	17.7 (1731)	16.3 (1559)	14.6 (476)
20 to <30 ethanol, g/d	9.9 (3569)	10.7 (608)	10.1 (794)	9.6 (938)	10.4 (997)	7.1 (232)
≥30 ethanol, g/d	10.5 (3801)	13.2 (750)	10.5 (828)	10.1 (984)	10.0 (956)	8.7 (283)
Age, y	49.1 ± 11.9	52.5 ± 10.1	48.4 ± 11.6	47.8 ± 11.8	50 ± 12.2	46.4 ± 13.1
BMI, kg/m^2^	25.6 ± 3.9	26.2 ± 4.1	25.5 ± 3.8	25.5 ± 3.9	25.5 ± 3.9	25.6 ± 4.1
Waist circumference, cm	85.0 ± 11.3	87.3 ± 11.8	84.8 ± 11.3	84.7 ± 11.2	84.1 ± 10.9	84.8 ± 11.7
Diastolic blood pressure, mm Hg	77.8 ± 10.6	79.1 ± 10.5	77.6 ± 10.4	77.4 ± 10.5	77.5 ± 10.7	77.3 ± 11.0
Systolic blood pressure, mm Hg	126.0 ± 18.7	128.88 ± 19.21	125.4 ± 18.4	124.9 ± 18.2	126.2 ± 19.1	125.0 ± 18.6
Total cholesterol, mmol/L	5.63 ± 1.15	5.84 ± 1.14	5.60 ± 1.13	5.57 ± 1.15	5.64 ± 1.16	5.5 ± 1.2
HDL cholesterol, mmol/L	1.42 ± 0.40	1.39 ± 0.41	1.43 ± 0.40	1.42 ± 0.39	1.45 ± 0.40	1.40 ± 0.38
Total-/HDL cholesterol, mmol/L	4.24 ± 3.59	4.44 ± 8.44	4.21 ± 1.49	4.21 ± 1.49	4.17 ± 1.44	4.23 ± 1.49
DHD15-index	66.1 ± 14.4	62.7 ± 14.5	65.7 ± 14.3	66.4 ± 14.2	68.3 ± 13.9	65.7 ± 14.7
Fruit, g/d	128 [160]	124 [182]	124 [173]	125 [158]	181 [165]	179 [173]
Pure fruit juice, g/d	40 [119]	0	12 [13]	44 [22]	131 [16]	267 [38]
Sugar-sweetened beverages,^5^ g/d	43 [106]	35 [104]	35 [95]	49 [101]	47 [102]	69 [154]
Dairy beverages, g/d	171 [315]	90 [233]	143 [256]	200 [354]	200 [342]	200 [351]
Coffee, g/d	450 [433]	450 [450]	450 [450]	450 [425]	360 [375]	338 [450]
Total energy intake, kcal/d	1956 [748]	1831 [781]	1916 [746]	1987 [734]	1953 [682]	2164 [806]

1 Values are percentages (frequencies), means ± SDs, or medians [IQR]; *n* = 36,147. DHD15-index, Dutch Healthy Diet Index 2015.

2 “glasses/wk” indicates glass(es) of 150 mL/wk.

3 Educational level categorized as “low” (primary education, lower vocational education, advanced elementary education), “intermediate” (intermediate vocational education, completion of first 3 y of higher general secondary education), or “high” (completed higher general secondary education, higher vocational education, and university).

4 Physical activity categorized as “inactive (sedentary job and no recreational activity) to moderately inactive (sedentary job with <0.5 h recreational activity/d or standing job with no recreational activity)” or "moderately active (sedentary job with 0.5–1 h recreational activity/d or standing job with 0.5 h recreational activity/d, or physical job with no recreational activity) to active (sedentary job with >1 h recreational activity/d or standing job with >0.5 h recreational activity/d, or physical job with at least some recreational activity or heavy manual job)".

5 Sugar-sweetened beverages included sugar-containing soft-drinks and fruit syrups.

### Pure fruit juice and type 2 diabetes

After adjustment for age and sex, compared with no consumption, pure fruit juice consumption of ≤8 glasses/wk was significantly associated with a reduced risk of type 2 diabetes incidence (**Table 2**, model 1). This association was attenuated and became nonsignificant after further adjustment for educational level, physical activity, smoking, family history of diabetes, alcohol consumption, coffee consumption, DHD15-index, and fruit consumption, with HRs (95% CIs) ranging from 0.92 (0.79, 1.09) to 1.03 (0.83, 1.26) across the categories of pure fruit juice consumption (Table 2, model 2). Adjustment for diet quality and educational level had the most impact on the association (**Supplemental Table 2**). The additional inclusion of possible intermediate factors yielded similar results (Table 2, models 3 and 4). No statistically significant interactions were found between pure fruit juice consumption and sex (*P* = 0.71), physical activity (*P* = 0.62), and BMI (*P* = 0.47) (model 2).

**TABLE 2:** HRs (95% CIs) for the association between pure fruit juice consumption and type 2 diabetes among 36,147 EPIC-NL participants^1^

	Categories of pure fruit juice consumption^2^					
	Nondrinkers	<1 glass/wk	1 to <4 glasses/wk	4 to <8 glasses/wk	≥8 glasses/wk	*P*-trend
All participants, *n*	5669	7874	9782	9561	3261	
Type 2 diabetes*, n*	305	299	363	379	131	
Mean follow-up period, y	14.4	14.7	14.7	14.6	14.5	
Model 1	1.00	0.84 (0.72, 0.99)	0.85 (0.73, 0.99)	0.81 (0.70, 0.94)	0.97 (0.79, 1.20)	0.59
Model 2	1.00	0.92 (0.79, 1.09)	0.95 (0.82, 1.11)	0.92 (0.79, 1.08)	1.03 (0.83, 1.26)	0.47
Model 3	1.00	0.94 (0.80, 1.11)	0.98 (0.84, 1.15)	0.95 (0.82, 1.11)	1.08 (0.88, 1.34)	0.22
Model 4	1.00	1.00 (0.85, 1.17)	0.98 (0.84, 1.14)	0.97 (0.84, 1.14)	0.98 (0.80, 1.21)	0.99
Participants with low fruit consumption, *n*	2534	3563	4255	3016	1089	
Type 2 diabetes*, n*	126	127	145	107	34	
Mean follow-up period, y	14.4	14.8	14.7	14.6	14.6	
Model 1	1.00	0.88 (0.69, 1.13)	0.88 (0.69, 1.12)	0.86 (0.66, 1.11)	0.91 (0.62, 1.33)	0.53
Model 2	1.00	0.94 (0.73, 1.21)	0.94 (0.74, 1.21)	0.93 (0.71, 1.21)	0.91 (0.62, 1.33)	0.53
Model 3	1.00	0.96 (0.74, 1.23)	0.97 (0.76, 1.25)	0.96 (0.74, 1.26)	0.96 (0.65, 1.41)	0.72
Model 4	1.00	0.99 (0.77, 1.27)	0.93 (0.73, 1.19)	0.93 (0.71, 1.21)	0.84 (0.57, 1.24)	0.20
Participants with high fruit consumption, *n*	3135	4311	5527	6545	2172	
Type 2 diabetes*, n*	179	172	218	272	97	
Mean follow-up period, y	14.4	14.7	14.7	14.6	14.5	
Model 1	1.00	0.81 (0.66, 1.00)	0.84 (0.69, 1.02)	0.80 (0.67, 0.97)	1.01 (0.79, 1.29)	0.18
Model 2	1.00	0.90 (0.74, 1.13)	0.97 (0.79, 1.19)	0.94 (0.78, 1.14)	1.11 (0.86, 1.43)	0.22
Model 3	1.00	0.91 (0.74, 1.13)	0.98 (0.80, 1.20)	0.95 (0.79, 1.16)	1.13 (0.87, 1.45)	0.11
Model 4	1.00	0.99 (0.81, 1.23)	1.00 (0.82, 1.22)	1.00 (0.82, 1.21)	1.03 (0.80, 1.32)	0.39

1 Values are HRs (95% CIs) unless otherwise indicated. Model 1 adjusted for age and sex. Model 2 adjusted for age, sex, educational level, physical activity, smoking, family history of diabetes, DHD15-index, alcohol, coffee, sugar-sweetened beverages, and fruit. Model 3 adjusted for age, sex, educational level, physical activity, smoking, family history of diabetes, DHD15-index, alcohol, coffee, sugar-sweetened beverages, fruit, and energy intake. Model 4 adjusted for age, sex, educational level, physical activity, smoking, family history of diabetes, DHD15-index, alcohol, coffee, sugar-sweetened beverages, fruit, BMI, and waist circumference. DHD15-index, Dutch Healthy Diet Index 2015; EPIC-NL, European Prospective Investigation into Cancer and Nutrition–Netherlands.

2 “glasses/wk” indicates glass(es) of 150 mL/wk.

### Associations in low and high fruit consumers

In both low fruit consumers and high fruit consumers no association was observed between pure fruit juice consumption and type 2 diabetes after adjustment for age, sex, educational level, physical activity, smoking, family history of diabetes, alcohol consumption, coffee consumption, DHD15-index, and fruit consumption. In low fruit consumers, HRs (95% CIs) ranged between 0.91 (0.62, 1.33) and 0.94 (0.73, 1.21) and in high fruit consumers ranged between 0.90 (0.74, 1.13) and 1.11 (0.86, 1.43) (Table 2, model 2). Formal testing for interaction showed that fruit consumption did not modify the association of pure fruit juice consumption with type 2 diabetes incidence (*P* = 0.68).

### Fruit consumption and type 2 diabetes

After adjustment for age and sex, compared with the lowest quintile of fruit consumption, the middle quintile (120–185 g/d) and the highest quintile (≥256 g/d) were statistically significantly associated with a lower incidence of type 2 diabetes (**Table 3**, model 1). This association was attenuated and became nonsignificant after further adjustment for educational level, physical activity, smoking, family history of diabetes, alcohol consumption, coffee consumption, DHD15-index, and fruit juice consumption, with HRs (95% CIs) ranging from 0.93 (0.78, 1.10) to 1.00 (0.84, 1.19) across the categories of pure fruit juice consumption (Table 2, model 2). Adjustment for diet quality resulted in the strongest attenuation (**Supplemental Table 3**). We found no statistically significant interactions between fruit consumption and sex (*P* = 0.94), physical activity (*P* = 0.83), and BMI (*P* = 0.16) (model 2). Additional inclusion of possible intermediate factors yielded similar results (Table 2, models 3 and 4).

**TABLE 3:** HRs (95% CIs) for the association between fruit consumption and type 2 diabetes among 36,147 EPIC-NL participants^1^

	Quintiles of fruit consumption					
	<69 g/d	69 to <121 g/d	121 to <186 g/d	186 to <259 g/d	≥259 g/d	*P*-trend
*n*	7228	7229	7230	7230	7230	
Type 2 diabetes*, n*	261	278	287	334	317	
Mean follow-up period, y	14,6	14,7	14,7	14,5	14,6	
Model 1	1.00	0.91 (0.77, 1.08)	0.84 (0.71, 1.00)	0.89 (0.76, 1.06)	0.82 (0.69, 0.97)	0.02
Model 2	1.00	0.99 (0.84, 1.18)	0.93 (0.78, 1.10)	1.00 (0.84, 1.19)	0.98 (0.82, 1.17)	0.66
Model 3	1.00	1.02 (0.86, 1.21)	0.95 (0.80, 1.13)	1.04 (0.87, 1.23)	1.03 (0.86, 1.23)	0.91
Model 4	1.00	1.03 (0.87, 1.22)	0.96 (0.80, 1.14)	1.04 (0.88, 1.24)	1.01 (0.85, 1.21)	0.96

1 Values are HRs (95% CIs) unless otherwise indicated. Model 1 adjusted for age and sex. Model 2 adjusted for age, sex, educational level, physical activity, smoking, family history of diabetes, DHD15-index, alcohol, coffee, sugar-sweetened beverages, and fruit juice. Model 3 adjusted for age, sex, educational level, physical activity, smoking, family history of diabetes, DHD15-index, alcohol, coffee, sugar-sweetened beverages, fruit juice, and energy intake. Model 4 adjusted for age, sex, educational level, physical activity, smoking, family history of diabetes, DHD15-index, alcohol, coffee, sugar-sweetened beverages, fruit juice, BMI, and waist circumference. DHD15-index, Dutch Healthy Diet Index 2015; EPIC-NL, European Prospective Investigation into Cancer and Nutrition–Netherlands.

### Sensitivity analyses

Including unverified potential diabetes cases showed higher point estimates for pure fruit juice consumption (**Supplemental Table 4**), whereas most point estimates for fruit consumption were slightly lower (**Supplemental Table 5**). For both pure fruit juice and fruit consumption all 95% CIs largely overlapped, and all adjusted associations remained nonsignificant. Excluding “other fruit juices” did not considerably change our findings (**Supplemental Table 6**). Separate analyses for apple juice showed higher point estimates for the 2 highest categories (4 to <8 glasses/wk and ≥8 glasses/wk), and separate analyses for orange/grapefruit juice showed higher point estimates for the highest category (≥8 glasses/wk), but all adjusted associations for both apple juice and orange/grape fruit juice remained nonsignificant (**Supplemental Tables 7** and **8**).

## Discussion

In this study, pure fruit juice consumption was not associated with the incidence of type 2 diabetes and this lack of an association did not differ between participants with a low and those with a high fruit consumption. Furthermore, we found no association between fruit consumption and the incidence of type 2 diabetes.

### Strengths and limitations

Strengths of this study are the prospective design, large sample size, and long follow-up period. Furthermore, we were able to adjust for many relevant confounders and we used verified diabetes cases in our study to minimize the chance of including false-positive diabetes cases and the possible attenuation of associations due to misclassification. Some potential limitations should be considered. The presence of diabetes may go undetected for ≤12 y before its clinical diagnosis (21). These undetected diabetes cases may have been misclassified as nondiabetics, possibly resulting in attenuation of existing associations. With regard to the assessment of the consumption of pure fruit juice, we cannot exclude that some participants, contrary to the intention of the questionnaire, reported not 100% fruit juice as pure fruit juice. This may have led to them being misclassified as pure fruit juice consumers. We assumed that misclassification of pure fruit juice consumption would not be related to type 2 diabetes and therefore exposure misclassification is likely to have been nondifferential and tend to attenuate the observed associations. The sensitivity analysis in which we excluded “other fruit juice,” because we assumed this category is most prone to misclassification, yielded similar results as the analysis that included this category. Furthermore, data on fruit juice and fruit consumption were collected at baseline and may have changed during follow-up. To assess the generalizability of our results to current consumption levels, we compared the pure fruit juice consumption level at study baseline with the Dutch National Food Consumption Survey 2007–2010 (22). Median pure fruit juice consumption in the 2007–2010 survey was similar (40 g/d) to the median consumption in our study population. Furthermore, the level of pure fruit juice consumption in our study was within the range observed in different countries worldwide in 2010 (23). Last, although we were able to adjust for many relevant confounders, it is possible that these covariates could have changed during follow-up. Therefore, residual confounding cannot be ruled out.

### Results of other studies

In line with our findings, a meta-analysis by Xi et al. (8) observed no association between pure fruit juice consumption and type 2 diabetes incidence for the highest versus lowest consumption category (RR: 1.03; 95% CI: 0.91, 1.18). This meta-analysis was exclusively based on consumption of 100% fruit juice and included 4 cohort studies. Two prospective cohort studies in women only, which were not included in this meta-analysis, also found no association between pure fruit juice consumption and type 2 diabetes (10, 11). Another prospective cohort study in women (9) showed that an increase of 1 serving/d (237 mL) of pure fruit juice consumption was associated with an increased hazard of diabetes (RR: 1.18; 95% CI: 1.10, 1.26). However, no adjustment was made for educational level, whereas our study showed that adjustment for educational level strongly attenuated the observed association. Very recently, another meta-analysis (24) was published that found no association between pure fruit juice and type 2 diabetes. Furthermore, a meta-analysis of 18 randomized controlled trials examining effects of pure fruit juice on glucose-insulin homeostasis (25) showed a neutral effect of pure fruit juice on glycemic control. This supports our finding that consumption of pure fruit juice is not associated with the risk of type 2 diabetes.

Two meta-analyses from 2014 reported on the association of fruit consumption categories with incidence of type 2 diabetes (1, 3). Both studies showed a lower risk of type 2 diabetes with fruit consumption. For participants in the highest category of fruit consumption compared with those in the lowest category, they both showed an RR of ∼0.93. However, adequate adjustment for diet quality was found in only one of all included studies in these meta-analyses, whereas our study showed that adjustment for diet quality strongly attenuated the observed association towards the null. Furthermore, very recently, in July 2019, a dose-response meta-analysis (24) was published that showed no clear association between fruit consumption and the incidence of type 2 diabetes, with an HR of 0.98 (95% CI: 0.97, 1.00).

### Interpretation of the present study

Our study showed that any crude association for both fruit juice and fruit consumption was attenuated by adjustment for healthy dietary habits. We found that pure fruit juice drinkers tended to have healthier dietary habits. In other studies, pure fruit juice consumption was also associated with better diet quality (26–29), which emphasizes the need for adequate adjustment for diet quality in research on the association between pure fruit juice consumption and type 2 diabetes.

The classification of pure fruit juice in the same category as SSBs, which make up a large proportion of all of sugar-containing beverages, in the Dutch dietary guidelines of 2015 (7) is based on the comparable sugar content and the expected unfavorable health effects of sugar intake. A recent dose-response meta-analysis (24) estimated that an increase of 1 serving/d of SSBs intake was associated with a 26% increase in the hazard of type 2 diabetes (HR: 1.26; 95% CI: 1.11, 1.43). Our study did not show an association between the highest category (≥8 glasses/wk) of pure fruit juice consumption and the incidence of type 2 diabetes. Apparently, pure fruit juice consumption seems to have a different association with the risk of type 2 diabetes incidence than do SSBs. There may be 2 possible explanations for this discrepancy. First, pure fruit juice has a similar energy density and sugar content as SSBs (30), but SSBs contain no favorable components such as polyphenols, which may be protective against the risk of type 2 diabetes (31). Second, pure fruit juices have low glycemic indices (e.g., on the glucose reference scale: apple juice, 36; orange juice, 50), whereas SSBs have medium glycemic indices (e.g., Coca-Cola, 63). Although the literature is not consistent, several large prospective cohort studies showed a positive association between glycemic index and glycemic load and the risk of type 2 diabetes (32–34).

In conclusion, in our study, pure fruit juice and fruit consumption was not independently associated with the risk of type 2 diabetes incidence. Our study therefore does not provide evidence for associations between pure fruit juice and fruit consumption and reduced diabetes risk. However, it also does not provide evidence for an association between high (≥8 glasses/wk) pure fruit juice consumption and increased diabetes risk.

## Supplementary Material

nxz340_Supplemental_Files

## Notes

This work is part of the research program Doctoral Grant for Teachers, project number 023.005.010, which is financed by the Netherlands Organization for Scientific Research (NWO). The EPIC-NL study was funded by the “Europe against Cancer” Program of the European Commission (DG SANCO); the Dutch Ministry of Health, Welfare, and Sports (VWS); the Netherlands Organization for Health Research and Development (ZonMW); and the World Cancer Research Fund (WCRF).

Author disclosures: The authors report no conflicts of interest. The funders had no role in the study design, data collection and analysis, decision to publish, or preparation of the manuscript.

Supplemental Tables 1–8 and Supplemental Figure 1 are available from the “Supplementary data” link in the online posting of the article and from the same link in the online table of contents at https://academic.oup.com/jn/.

Abbreviations used: DHD15-index, Dutch Healthy Diet Index 2015; EPIC, European Prospective Investigation into Cancer and Nutrition; EPIC-NL, European Prospective Investigation into Cancer and Nutrition–Netherlands; FFQ, food-frequency questionnaire; SSB, sugar-sweetened beverage.
