# Supplementary material for: Pure Fruit Juice and Fruit Consumption Are Not Associated with Incidence of Type 2 Diabetes after Adjustment for Overall Dietary Quality in the European Prospective Investigation into Cancer and Nutrition–Netherlands (EPIC-NL) Study
Source: J Nutr. 2020 Jan 14;150(6):1470–7. doi: 10.1093/jn/nxz340 (PMC7269751; doi:10.1093/jn/nxz340)
Supplement: nxz340_Supplemental_Files [file nxz340_supplemental_files.zip › Online Supplementary Material_rev2_DEF.docx]

JN-2019-1098

**Pure fruit juice and fruit consumption are not associated with incidence of type 2 diabetes after adjustment for overall dietary quality in the EPIC-NL Study.**

Scheffers et al.

“Online Supplementary Material”

| Supplemental table 1. Baseline characteristics according to quintiles of fruit consumption (*n* = 36,147)^1^ | | | | |  |  |  |  |  |  |
| --- | --- | --- | --- | --- | --- | --- | --- | --- | --- | --- |
|  |  |  |  |  |  |  |  |  |  |  |
|  | Quintiles of fruit consumption | | | | |  |  |  |  |  |
|  | <69 g/day | 69-<121 g/day | 121-<186 g/day | 186-<259 g/day | ≥259 g/day |  |  |  |  |  |
|  | (*n* = 7228) | (*n* = 7229) | (*n* = 7230) | (*n* = 7230) | (*n* = 7230) |  |  |  |  |  |
| Cohort |  |  |  |  |  |  |  |  |  |  |
| Prospect | 22.8 (1650) | 35.5 (2569) | 44.0 (3178) | 54.3 (3926) | 60.8 (4393) |  |  |  |  |  |
| MORGEN | 77.2 (5578) | 64.5 (4660) | 56.0 (4052) | 45.7 (3304) | 39.2 (2837) |  |  |  |  |  |
| Sex |  |  |  |  |  |  |  |  |  |  |
| male | 43.3 (3132) | 28.0 (2024) | 24.7 (1786) | 17.6 (1274) | 13.9 (1008) |  |  |  |  |  |
| Education level^2^ |  |  |  |  |  |  |  |  |  |  |
| Low | 58.5 (4230) | 56.7 (4100) | 57.1 (4125) | 59.5 (4305) | 56.0 (4050) |  |  |  |  |  |
| Intermediate | 25.1 (1815) | 22.7 (1644) | 21.1 (1525) | 20.7 (1498) | 20.0 (1445) |  |  |  |  |  |
| High | 16.4 (1183) | 20.5 (1485) | 21.9 (1580) | 19.7 (1427) | 24.0 (1735) |  |  |  |  |  |
| Family history of diabetes |  |  |  |  |  |  |  |  |  |  |
| None | 77.5 (5600) | 78.0 (5638) | 76.9 (5561) | 75.2 (5433) | 76.0 (5493) |  |  |  |  |  |
| One parent | 15.0 (1082) | 15.9 (1149) | 16.9 (1224) | 19.0 (1374) | 18.4 (1327) |  |  |  |  |  |
| Both parents | 0.7 (49) | 0.84 (61) | 0.8 (57) | 1.2 (89) | 1.0 (70) |  |  |  |  |  |
| Unknown | 6.9 (497) | 5.3 (381) | 5.4 (388) | 4.6 (334) | 4.7 (340) |  |  |  |  |  |
| Smoking status |  |  |  |  |  |  |  |  |  |  |
| never | 28.7 (2076) | 36.5 (2641) | 39.6 (2865) | 40.8 (2950) | 45.1 (3260) |  |  |  |  |  |
| former | 24.1 (1741) | 29.4 (2127) | 32.0 (2314) | 35.6 (2573) | 35.7 (2583) |  |  |  |  |  |
| current | 47.2 (3411) | 34.0 (2461) | 28.4 (2051) | 23.6 (1707) | 19.2 (1387) |  |  |  |  |  |
| Physical activity^3^ |  |  |  |  |  |  |  |  |  |  |
| Inactive to moderately inactive | 37.2 (2690) | 32.8 (2372) | 31.6 (2282) | 30.4 (2197) | 27.9 (2015) |  |  |  |  |  |
| moderately active to active | 62.8 (4538) | 67.2 (4857) | 68.4 (4948) | 69.6 (5033) | 72.1 (5215) |  |  |  |  |  |
| Alcohol intake |  |  |  |  |  |  |  |  |  |  |
| never | 0.6 (43) | 0.33 (24) | 0.5 (35) | 0.4 (32) | 0.4 (31) |  |  |  |  |  |
| < 10 ethanol (g/day) | 55.9 (4042) | 61.1 (44.2) | 63.2 (4566) | 66.4 (4798) | 66.9 (4836) |  |  |  |  |  |
| 10 - < 20 ethanol (g/day) | 16.1 (1161) | 17.4 (1257) | 16.7 (1207) | 16.3 (1177) | 15.9 (1152) |  |  |  |  |  |
| 20 - < 30 ethanol (g/day) | 10.5 (756) | 10.6 (764) | 10.3 (741) | 9.0 (647) | 9.1 (661) |  |  |  |  |  |
| ≥ 30 ethanol (g/day) | 17.0 (1226) | 10.6 (768) | 9.4 (681) | 8.0 (576) | 7.6 (550) |  |  |  |  |  |
|  |  |  |  |  |  |  |  |  |  |  |
| Age (years) | 44.4 ± 12.0 | 47.5 ± 12.0 | 49.4 ± 11.8 | 51.8 ± 11.0 | 52.6 ± 10.7 |  |  |  |  |  |
| BMI | 25.5 ± 4.1 | 25.4 ± 3.9 | 25.6 ± 3.9 | 25.8 ± 3.9 | 25.7 ± 3.9 |  |  |  |  |  |
| Waist-circumference (cm) | 86.4 ± 12.3 | 84.8 ± 11.5 | 85.0 ± 11.3 | 84.7 ± 10.9 | 83.9 ± 10.6 |  |  |  |  |  |
| Diastolic blood pressure (mm Hg) | 77.4 ± 10.7 | 77.3 ± 10.7 | 77.8 ± 10.6 | 78.0 ± 10.5 | 78.1 ± 10.5 |  |  |  |  |  |
| Systolic blood pressure (mm Hg) | 123.4 ± 17.7 | 124.5 ± 18.5 | 126.1 ± 18.9 | 127.8 ± 19.0 | 128.1 ± 19.1 |  |  |  |  |  |
| Total cholesterol (mmol/L) | 5.46 ± 1.12 | 5.55 ± 1.16 | 5.62 ± 1.17 | 5.75 ± 1.13 | 5.77 ± 1.15 |  |  |  |  |  |
| HDL-cholesterol (mmol/L) | 1.34 ± 0.39 | 1.41 ± 0.39 | 1.42 ± 0.39 | 1.45 ± 0.40 | 1.48 ± 0.40 |  |  |  |  |  |
| Total-/HDL cholesterol (mmol/L) | 4.38 ± 1.61 | 4.23 ± 1.54 | 4.16 ± 7.45 | 4.25 ± 1.45 | 4.18 ± 1.42 |  |  |  |  |  |
| DHD15-index | 59.6 ± 13.9 | 65.1 ± 13.8 | 66.2 ± 13.8 | 68.4 ± 13.7 | 71.1 ± 13.9 |  |  |  |  |  |
|  |  |  |  |  |  |  |  |  |  |  |
| Fruit (g/day) | 35 [36] | 106 [31] | 128 [52] | 240 [14] | 361 [114] |  |  |  |  |  |
| Fruit juice (g/day) | 26 [73] | 40 [107] | 47 [122] | 53 [122] | 68 [116] |  |  |  |  |  |
| Sugar-sweetened beverages (g/day)^4^ | 69 [142] | 53 [112] | 43 [101] | 35 [91] | 34 [78] |  |  |  |  |  |
| Dairy beverages (g/day) | 115 [268] | 190 [361] | 173 [308] | 200 [336] | 200 [360] |  |  |  |  |  |
| Coffee (g/day) | 450 [450] | 450 [450] | 450 [405] | 450 [353] | 360 [387] |  |  |  |  |  |
| Total energy intake (kcal/day) | 2056 [874] | 1975 [761] | 1956 [739] | 1896 [686] | 1915 [654] |  |  |  |  |  |
| ^1^Values are percentages (frequencies), means ± SDs, or medians [IQR]. DHD15-index: Dutch Healty Diet index 2015. | | | | |  |  |  |  |  |  |
| ^2^Education level categorized as "low" (primary education, lower vocational education, advanced elementary education), "intermediate" (intermediate vocational education, | | | | | | | | |  |  |
| completion of first three years of higher general secondary education), or "high" (completed higher general secondary education, higher vocational education and university). | | | | | | | | |  |  |
| ^3^Physical activity categorized as "inactive (sedentary job and no recreational activity) to moderately inactive (sedentary job with <0.5h recreational activity per day or standing job with no recreational activity)" | | | | | | | | | | |
| or "moderately active (sedentary job with 0.5 to 1h recreational activity per day or standing job with 0.5h recreational activity per day or physical job with no recreational activity) | | | | | | | | |  |  |
| to active (sedentary job with >1h recreational activity per day or standing job with >0.5h recreational activity per day or physical job with at least some recreational activity or heavy manual job)" | | | | | | | | | | |
| ^4^Sugar-sweetened beverages included sugar-containing soft-drinks and Roosvicee/Karvan cevitam. | | | |  |  |  |  |  |  |  |
|  |  |  |  |  |  |  |  |  |  |  |

| Supplemental table 2. HRs and 95% CIs for the association between fruit juice consumption and type 2 diabetes adjusted for individual potential confounders^1^ | | | | | |  |
| --- | --- | --- | --- | --- | --- | --- |
|  |  |  |  |  |  |  |
|  | Categories of fruit juice consumption | | | | |  |
|  | non-drinkers | <1 gl/wk^2^ | 1-<4 gl/wk^2^ | 4-<8 gl/wk^2^ | ≥8 gl/wk^2^ |  |
|  |  |  |  |  |  |  |
| Model 1^3^ | 1.00 | 0.84 (0.72 - 0.99) | 0.85 (0.73 - 0.99) | 0.81 (0.70 - 0.94) | 0.97 (0.79 - 1.20) |  |
| Model 1 + adjustment for educational level | 1.00 | 0.90 (0.76 - 1.05) | 0.91 (0.78 - 1.06) | 0.86 (0.74 - 1.01) | 1.02 (0.83 - 1.26) |  |
| Model 1 + adjustment for pshysical activity | 1.00 | 0.85 (0.72 - 1.00) | 0.86 (0.74 - 1.01) | 0.82 (0.71 - 0.96) | 0.99 (0.80 - 1.21) |  |
| Model 1 + adjustment for smoking | 1.00 | 0.85 (0.72 - 1.00) | 0.86 (0.74 - 1.01) | 0.82 (0.71 - 0.96) | 0.99 (0.80 - 1.22) |  |
| Model 1 + adjustment for family history of diabetes | 1.00 | 0.85 (0.72 - 0.99) | 0.87 (0.74 - 1.01) | 0.82 (0.71 - 0.96) | 0.99 (0.80 - 1.21) |  |
| Model 1 + adjustment for alcohol | 1.00 | 0.83 (0.71 - 0.98) | 0.85 (0.73 - 0.99) | 0.81 (0.70 - 0.94) | 0.95 (0.77 - 1.16) |  |
| Model 1 + adjustment for DHD15-index | 1.00 | 0.88 (0.75 - 1.03) | 0.90 (0.77 - 1.04) | 0.86 (0.74 - 1.01) | 1.02 (0.83 - 1.25) |  |
| Model 1 + adjustment for coffee | 1.00 | 0.84 (0.72 - 0.99) | 0.86 (0.73 - 1.00) | 0.82 (0.70 - 0.95) | 0.98 (0.80 - 1.21) |  |
| Model 1 + adjustment for sugar sweetened beverages | 1.00 | 0.85 (0.72 - 0.99) | 0.85 (0.73 - 0.99) | 0.80 (0.69 - 0.93) | 0.93 (0.76 - 1.14) |  |
| Model 1 + adjustment for fruit | 1.00 | 0.84 (0.72 - 0.99) | 0.86 (0.74 - 1.00) | 0.83 (0.71 - 0.96) | 1.00 (0.81 - 1.22) |  |
| ^1^Values are HRs (95% CIs). |  |  |  |  |  |  |
| ^2^gl/wk = glass(es) of 150 mL per week. |  |  |  |  |  |  |
| ^3^adjusted for age and sex. |  |  |  |  |  |  |
|  |  |  |  |  |  |  |

| Supplemental table 3. HRs and 95% CIs for the association between fruit consumption and type 2 diabetes adjusted for individual potential confounders^1^ | | | | | |  |
| --- | --- | --- | --- | --- | --- | --- |
|  |  |  |  |  |  |  |
|  | Quintiles of fruit consumption | | | | |  |
|  | <69 g/day | 69-<121 g/day | 121-<186 g/day | 186-<259 g/day | ≥259 g/day |  |
|  |  |  |  |  |  |  |
| Model 1^2^ | 1.00 | 0.91 (0.77 - 1.08) | 0.84 (0.71 - 1.00) | 0.89 (0.76 - 1.06) | 0.82 (0.69 - 0.97) |  |
| Model 1 + educational level | 1.00 | 0.94 (0.80 - 1.12) | 0.88 (0.74 - 1.04) | 0.95 (0.80 - 1.12) | 0.89 (0.75 - 1.06) |  |
| Model 1 + pshysical activity | 1.00 | 0.93 (0.78 - 1.10) | 0.86 (0.72 - 1.02) | 0.92 (0.78 - 1.08) | 0.84 (0.71 - 1.00) |  |
| Model 1 + smoking | 1.00 | 0.92 (0.77 - 1.09) | 0.85 (0.72 - 1.01) | 0.91 (0.76 - 1.07) | 0.83 (0.70 - 0.99) |  |
| Model 1 + family history of diabetes | 1.00 | 0.92 (0.78 - 1.09) | 0.85 (0.72 - 1.01) | 0.89 (0.76 - 1.06) | 0.82 (0.69 - 0.98) |  |
| Model 1 + alcohol consumption | 1.00 | 0.89 (0.75 - 1.06) | 0.81 (0.69 - 0.97) | 0.86 (0.73 - 1.02) | 0.79 (0.66 - 0.93) |  |
| Model 1 + DHD15-index | 1.00 | 0.97 (0.82 - 1.15) | 0.90 (0.76 - 1.07) | 0.99 (0.83 - 1.17) | 0.93 (0.79 - 1.11) |  |
| Model 1 + adjustment for coffee | 1.00 | 0.92 (0.77 - 1.09) | 0.84 (0.71 - 1.00) | 0.90 (0.76 - 1.07) | 0.83 (0.70 - 0.98) |  |
| Model 1 + adjustment for sugar sweetened beverages | 1.00 | 0.92 (0.78 - 1.09) | 0.85 (0.72 - 1.01) | 0.91 (0.77 - 1.08) | 0.84 (0.71 - 0.99) |  |
| Model 1 + fruit juice consumption | 1.00 | 0.91 (0.77 - 1.08) | 0.84 (0.70 - 0.99) | 0.89 (0.75 - 1.05) | 0.81 (0.68 - 0.96) |  |
|  |  |  |  |  |  |  |
| ^1^Values are HRs (95% CIs). |  |  |  |  |  |  |
| ^2^adjusted for age and sex. |  |  |  |  |  |  |
|  |  |  |  |  |  |  |

| Supplemental table 4. Sensitivity analyses - HRs and 95% CIs for the association between fruit juice consumption and type 2 diabetes based on additional added (unverified) diabetes cases^1^ | | | | | | | | | | |
| --- | --- | --- | --- | --- | --- | --- | --- | --- | --- | --- |
|  |  |  |  |  |  |  |  |  |  |  |
|  | Categories of fruit juice consumption | | | | |  |  |  |  |  |
|  | non-drinkers | <1 gl/wk^2^ | 1-<4 gl/wk^2^ | 4-<8 gl/wk^2^ | ≥8 gl/wk^2^ | *P*-trend |  |  |  |  |
|  |  |  |  |  |  |  |  |  |  |  |
| All participants, *n* | 5755 | 7995 | 9880 | 9688 | 3319 |  |  |  |  |  |
| *type 2 diabetes, n* | 391 | 420 | 461 | 506 | 189 |  |  |  |  |  |
| Mean follow-up period, y | 14.4 | 14.7 | 14.7 | 14.6 | 14.5 |  |  |  |  |  |
| Model 1^3^ | 1.00 | 1.02 (0.89 - 1.18) | 1.08 (0.94 - 1.23) | 0.98 (0.85 - 1.11) | 1.11 (0.93 - 1.33) | 0.47 |  |  |  |  |
| Model 2^4^ | 1.00 | 1.01 (0.88 - 1.17) | 1.09 (0.95 - 1.25) | 0.97 (0.84 - 1.11) | 1.10 (0.92 - 1.31) | 0.64 |  |  |  |  |
| Model 3^5^ | 1.00 | 1.01 (0.88 - 1.16) | 1.08 (0.94 - 1.24) | 0.96 (0.84 - 1.10) | 1.08 (0.90 - 1.30) | 0.76 |  |  |  |  |
| Model 4^6^ | 1.00 | 1.06 (0.92 - 1.22) | 1.09 (0.95 - 1.25) | 0.99 (0.86 - 1.13) | 1.04 (0.87 - 1.24) | 0.75 |  |  |  |  |
|  |  |  |  |  |  |  |  |  |  |  |
| Participants with low fruit consumption, *n* | 2568 | 3618 | 4294 | 3056 | 1103 |  |  |  |  |  |
| *type 2 diabetes, n* | 160 | 182 | 184 | 147 | 48 |  |  |  |  |  |
| Mean follow-up period, y | 14.4 | 14.8 | 14.7 | 14.6 | 14.6 |  |  |  |  |  |
| Model 1^3^ | 1.00 | 1.01 (0.81 - 1.25) | 1.10 (0.88 - 1.36) | 0.99 (0.79 - 1.24) | 1.34 (0.96 - 1.85) | 0.24 |  |  |  |  |
| Model 2^4^ | 1.00 | 0.93 (0.75 - 1.16) | 1.07 (0.86 - 1.34) | 0.95 (0.76 - 1.20) | 1.30 (0.93 - 1.80) | 0.16 |  |  |  |  |
| Model 3^5^ | 1.00 | 0.93 (0.74 - 1.16) | 1.06 (0.85 - 1.33) | 0.95 (0.75 - 1.19) | 1.28 (0.92 - 1.79) | 0.18 |  |  |  |  |
| Model 4^6^ | 1.00 | 1.03 (0.83 - 1.29) | 1.06 (0.85 - 1.33) | 0.98 (0.77 - 1.23) | 1.16 (0.83 - 1.62) | 0.94 |  |  |  |  |
|  |  |  |  |  |  |  |  |  |  |  |
| Participants with high fruit consumption, *n* | 3187 | 4377 | 5586 | 6632 | 2216 |  |  |  |  |  |
| *type 2 diabetes, n* | 231 | 238 | 277 | 359 | 141 |  |  |  |  |  |
| Mean follow-up period, y | 14.4 | 14.7 | 14.6 | 14.6 | 14.5 |  |  |  |  |  |
| Model 1^3^ | 1.00 | 1.03 (0.86 - 1.24) | 1.06 (0.89 - 1.26) | 0.96 (0.82 - 1.14) | 1.06 (0.86 - 1.31) | 0.78 |  |  |  |  |
| Model 2^4^ | 1.00 | 1.06 (0.88 - 1.28) | 1.09 (0.91 - 1.30) | 0.96 (0.81 - 1.14) | 1.04 (0.84 - 1.30) | 0.73 |  |  |  |  |
| Model 3^5^ | 1.00 | 1.06 (0.88 - 1.27) | 1.08 (0.90 - 1.29) | 0.95 (0.80 - 1.12) | 1.02 (0.82 - 1.28) | 0.60 |  |  |  |  |
| Model 4^6^ | 1.00 | 1.05 (0.87 - 1.26) | 1.07 (0.89 - 1.28) | 0.97 (0.82 - 1.15) | 0.97 (0.78 - 1.21) | 0.60 |  |  |  |  |
|  |  |  |  |  |  |  |  |  |  |  |
| ^1^Values are HRs (95% CIs). |  |  |  |  |  |  |  |  |  |  |
| ^2^gl/wk = glass(es) of 150 mL per week. |  |  |  |  |  |  |  |  |  |  |
| ^3^adjusted for age and sex. |  |  |  |  |  |  |  |  |  |  |
| ^4^adjusted for age, sex, educational level, physical activity, smoking, family history of diabetes, DHD15-index, alcohol, coffee, sugar-sweetened beverages, fruit. | | | | | |  |  |  |  |  |
| ^5^adjusted for age, sex, educational level, physical activity, smoking, family history of diabetes, DHD15-index, alcohol, coffee, sugar-sweetened beverages, fruit and energy intake. | | | | | | | |  |  |  |
| ^6^adjusted for age, sex, educational level, physical activity, smoking, family history of diabetes, DHD15-index, alcohol, coffee, sugar-sweetened beverages, fruit, BMI and waist circumference. | | | | | | | | |  |  |
|  |  |  |  |  |  |  |  |  |  |  |
|  |  |  |  |  |  |  |  |  |  |  |

|  |  |  |  |  |  |  |  |  |  |  |  |
| --- | --- | --- | --- | --- | --- | --- | --- | --- | --- | --- | --- |
| Supplemental table 5. Sensitivity analyses - HRs and 95% CIs for the association between fruit consumption and type 2 diabetes based on additional added (unverified) diabetes cases^1^. | | | | | | | | | | | |
|  |  |  |  |  |  |  |  |  |  |  |  |
|  | Quintiles of fruit consumption | | | | |  |  |  |  |  |  |
|  | <69 g/day | 69-<121 g/day | 121-<186 g/day | 186-<259 g/day | ≥259 g/day | *P*-trend |  |  |  |  |  |
|  |  |  |  |  |  |  |  |  |  |  |  |
| *n* | 7329 | 7310 | 7325 | 7332 | 7341 |  |  |  |  |  |  |
| *type 2 diabetes, n* | 362 | 359 | 382 | 436 | 428 |  |  |  |  |  |  |
| Mean follow-up period, y | 14.6 | 14.7 | 14.7 | 14.5 | 14.6 |  |  |  |  |  |  |
| Model 1^2^ | 1.00 | 0.89 (0.77 - 1.03) | 0.85 (0.73 - 0.98) | 0.98 (0.85 - 1.13) | 0.88 (0.76 - 1.01) | 0.26 |  |  |  |  |  |
| Model 2^3^ | 1.00 | 0.95 (0.82 - 1.10) | 0.89 (0.77 - 1.04) | 1.03 (0.89 - 1.19) | 0.96 (0.82 - 1.12) | 0.98 |  |  |  |  |  |
| Model 3^4^ | 1.00 | 0.95 (0.82 - 1.10) | 0.89 (0.76 - 1..03) | 1.02 (0.88 - 1.18) | 0.95 (0.81 - 1.11) | 0.86 |  |  |  |  |  |
| Model 4^5^ | 1.00 | 0.98 (0.84 - 1.14) | 0.94 (0.81 - 1.10) | 1.05 (0.90 - 1.21) | 0.99 (0.85 - 1.15) | 0.87 |  |  |  |  |  |
|  |  |  |  |  |  |  |  |  |  |  |  |
| ^1^Values are HRs (95% CIs). |  |  |  |  |  |  |  |  |  |  |  |
| ^2^adjusted for age and sex. |  |  |  |  |  |  |  |  |  |  |  |
| ^3^adjusted for age, sex, educational level, physical activity, smoking, family history of diabetes, DHD15-index, alcohol, coffee, sugar-sweetened beverages, fruit juice. | | | | | | | |  |  |  |  |
| ^4^adjusted for age, sex, educational level, physical activity, smoking, family history of diabetes, DHD15-index, alcohol, coffee, sugar-sweetened beverages, fruit juice and energy intake. | | | | | | | | |  |  |  |
| ^5^adjusted for age, sex, educational level, physical activity, smoking, family history of diabetes, DHD15-index, alcohol, coffee, sugar-sweetened beverages, fruit juice, BMI and waist circumference. | | | | | | | | | |  |  |
|  |  |  |  |  |  |  |  |  |  |  |  |

| Supplemental table 6. HRs and 95% CIs for the association between pure fruit juice consumption and type 2 diabetes (without "other fruit juice") | | | | | | | |  |  |
| --- | --- | --- | --- | --- | --- | --- | --- | --- | --- |
|  |  |  |  |  |  |  |  |  |  |
|  | Categories of pure fruit juice consumption | | | | | |  |  |  |
|  | non-drinkers | <1 gl/wk^2^ | 1-<4 gl/wk^2^ | 4-<8 gl/wk^2^ | ≥8 gl/wk^2^ | *P*-trend |  |  |  |
|  |  |  |  |  |  |  |  |  |  |
| *n* | 5669 | 8890 | 10632 | 8367 | 2589 |  |  |  |  |
| *type 2 diabetes, n* | 305 | 342 | 403 | 318 | 109 |  |  |  |  |
| Mean follow-up period, y | 14.4 | 14.7 | 14.7 | 14.6 | 14.4 |  |  |  |  |
| Model 1^3^ | 1.00 | 0.84 (0.72 - 0.99) | 0.87 (0.75 - 1.01) | 0.77 (0.66 - 0.90) | 1.05 (0.84 - 1.31) | 0.58 |  |  |  |
| Model 2^4^ | 1.00 | 0.93 (0.80 - 1.09) | 0.98 (0.84 - 1.14) | 0.87 (0.74 - 1.03) | 1.10 (0.88 - 1.37) | 0.42 |  |  |  |
| Model 3^5^ | 1.00 | 0.95 (0.81 - 1.11) | 1.01 (0.87 - 1.18) | 0.90 (0.77 - 1.06) | 1.16 (0.93 - 1.45) | 0.21 |  |  |  |
| Model 4^6^ | 1.00 | 1.00 (0.85 - 1.17) | 1.01 (0.87 - 1.18) | 0.92 (0.78 - 1.08) | 1.04 (0.84 - 1.30) | 0.98 |  |  |  |
|  |  |  |  |  |  |  |  |  |  |
| ^1^Values are HRs (95% CIs). |  |  |  |  |  |  |  |  |  |
| ^2^gl/wk = glass(es) of 150 mL per week. | |  |  |  |  |  |  |  |  |
| ^3^adjusted for age and sex. |  |  |  |  |  |  |  |  |  |
| ^4^adjusted for age, sex, educational level, physical activity, smoking, family history of diabetes, DHD15-index, alcohol, coffee, sugar-sweetened beverages, fruit. | | | | | | |  |  |  |
| ^5^adjusted for age, sex, educational level, physical activity, smoking, family history of diabetes, DHD15-index, alcohol, coffee, sugar-sweetened beverages, fruit and energy intake. | | | | | | | | |  |
| ^6^adjusted for age, sex, educational level, physical activity, smoking, family history of diabetes, DHD15-index, alcohol, coffee, sugar-sweetened beverages, fruit, BMI and waist circumference. | | | | | | | | | |
|  |  |  |  |  |  |  |  |  |  |

| Supplemental table 7. HRs and 95% CIs for the association between pure apple juice consumption and type 2 diabetes among 36,147 EPIC-NL participants^1^ | | | | | | | | |  |
| --- | --- | --- | --- | --- | --- | --- | --- | --- | --- |
|  |  |  |  |  |  |  |  |  |  |
|  | Categories of pure apple juice consumption | | | | | |  |  |  |
|  | non-drinkers | < 1 gl/wk^2^ | 1-<4 gl/wk^2^ | 4-<8 gl/wk^2^ | ≥8 gl/wk^2^ | *P*-trend |  |  |  |
|  |  |  |  |  |  |  |  |  |  |
| *n* | 5669 | 21778 | 7337 | 1090 | 273 |  |  |  |  |
| *type 2 diabetes, n* | 305 | 844 | 268 | 45 | 15 |  |  |  |  |
| Mean follow-up period, y | 14.4 | 14.7 | 14.6 | 14.6 | 14.4 |  |  |  |  |
| Model 1^3^ | 1.00 | 0.84 (0.73 - 0.96) | 0.84 (0.71 - 0.99) | 0.96 (0.70 - 1.32) | 1.38 (0.82 - 2.31) | 0.21 |  |  |  |
| Model 2^4^ | 1.00 | 0.94 (0.82 - 1.07) | 0.93 (0.79 - 1.10) | 0.99 (0.73 - 1.36) | 1.78 (0.70 - 1.98) | 0.47 |  |  |  |
| Model 3^5^ | 1.00 | 0.96 (0.84 - 1.10) | 0.98 (0.82 - 1.16) | 1.05 (0.76 - 1.44) | 1.25 (0.74 - 2.10) | 0.26 |  |  |  |
| Model 4^6^ | 1.00 | 0.99 (0.87 - 1.13) | 0.95 (0.80 - 1.12) | 1.02 (0.74 - 1.39) | 0.93 (0.55 - 1.39) | 0.95 |  |  |  |
|  |  |  |  |  |  |  |  |  |  |
| ^1^Values are HRs (95% CIs). |  |  |  |  |  |  |  |  |  |
| ^2^gl/wk = glass(es) of 150 mL per week. | |  |  |  |  |  |  |  |  |
| ^3^adjusted for age and sex. |  |  |  |  |  |  |  |  |  |
| ^4^adjusted for age, sex, educational level, physical activity, smoking, family history of diabetes, DHD15-index, alcohol, coffee, sugar-sweetened beverages, fruit. | | | | | | | |  |  |
| ^5^adjusted for age, sex, educational level, physical activity, smoking, family history of diabetes, DHD15-index, alcohol, coffee, sugar-sweetened beverages, fruit and energy intake. | | | | | | | | |  |
| ^6^adjusted for age, sex, educational level, physical activity, smoking, family history of diabetes, DHD15-index, alcohol, coffee, sugar-sweetened beverages, fruit, BMI and waist circumference. | | | | | | | | | |
|  |  |  |  |  |  |  |  |  |  |

| Supplemental table 8. HRs and 95% CIs for the association between pure orange/grapefruit juice consumption and type 2 diabetes among 36,147 EPIC-NL participants^1^ | | | | | | | | | |  |
| --- | --- | --- | --- | --- | --- | --- | --- | --- | --- | --- |
|  |  |  |  |  |  |  |  |  |  |  |
|  | Categories of pure citrus juice consumption | | | | | |  |  |  |  |
|  | non-drinkers | <1 gl/wk^2^ | 1-<4 gl/wk^2^ | 4-<8 gl/wk^2^ | ≥8 gl/wk^2^ | *P*-trend |  |  |  |  |
|  |  |  |  |  |  |  |  |  |  |  |
| *n* | 5669 | 11842 | 11311 | 5985 | 1340 |  |  |  |  |  |
| *type 2 diabetes, n* | 305 | 467 | 405 | 239 | 61 |  |  |  |  |  |
| Mean follow-up period, y | 14.4 | 14.7 | 14.7 | 14.6 | 14.3 |  |  |  |  |  |
| Model 1 | 1.00 | 0.86 (0.74 - 0.99) | 0.84 (0.72 - 0.97) | 0.80 (0.67 - 0.94) | 1.14 (0.86 - 1.50) | 0.96 |  |  |  |  |
| Model 2 | 1.00 | 0.94 (0.81 - 1.08) | 0.94 (0.81 - 1.10) | 0.91 (0.76 - 1.08) | 1.18 (0.89 - 1.55) | 0.51 |  |  |  |  |
| Model 3 | 1.00 | 0.96 (0.83 - 1.11) | 0.98 (0.84 - 1.14) | 0.94 (0.79 - 1.12) | 1.24 (0.94 - 1.65) | 0.31 |  |  |  |  |
| Model 4 | 1.00 | 1.00 (0.86 - 1.15) | 0.98 (0.85 - 1.15) | 0.93 (0.79 - 1.11) | 1.10 (0.83 - 1.45) | 0.99 |  |  |  |  |
|  |  |  |  |  |  |  |  |  |  |  |
| ^1^Values are HRs (95% CIs). |  |  |  |  |  |  |  |  |  |  |
| ^2^gl/wk = glass(es) of 150 mL per week. | |  |  |  |  |  |  |  |  |  |
| ^3^adjusted for age and sex. |  |  |  |  |  |  |  |  |  |  |
| ^4^adjusted for age, sex, educational level, physical activity, smoking, family history of diabetes, DHD15-index, alcohol, coffee, sugar-sweetened beverages, fruit. | | | | | | | |  |  |  |
| ^5^adjusted for age, sex, educational level, physical activity, smoking, family history of diabetes, DHD15-index, alcohol, coffee, sugar-sweetened beverages, fruit and energy intake. | | | | | | | | |  |  |
| ^6^adjusted for age, sex, educational level, physical activity, smoking, family history of diabetes, DHD15-index, alcohol, coffee, sugar-sweetened beverages, fruit, BMI and waist circumference. | | | | | | | | | |  |
|  |  |  |  |  |  |  |  |  |  |  |
