# Supplementary material for: Pure Fruit Juice and Fruit Consumption Are Not Associated with Incidence of Type 2 Diabetes after Adjustment for Overall Dietary Quality in the European Prospective Investigation into Cancer and Nutrition–Netherlands (EPIC-NL) Study
Source: J Nutr. 2020 Jan 14;150(6):1470–7. doi: 10.1093/jn/nxz340 (PMC7269751; doi:10.1093/jn/nxz340)
Supplement: nxz340_Supplemental_Files [file nxz340_supplemental_files.zip › Supplemental figure 1_fruit juice_DM_JN_v2.pdf]

JN-2019-1098

Pure fruit juice and fruit consumption are not associated with incidence of type 2 diabetes after adjustment for overall dietary quality in the EPIC-NL Study.

Scheffers et al.

"Online Supplementary Material"

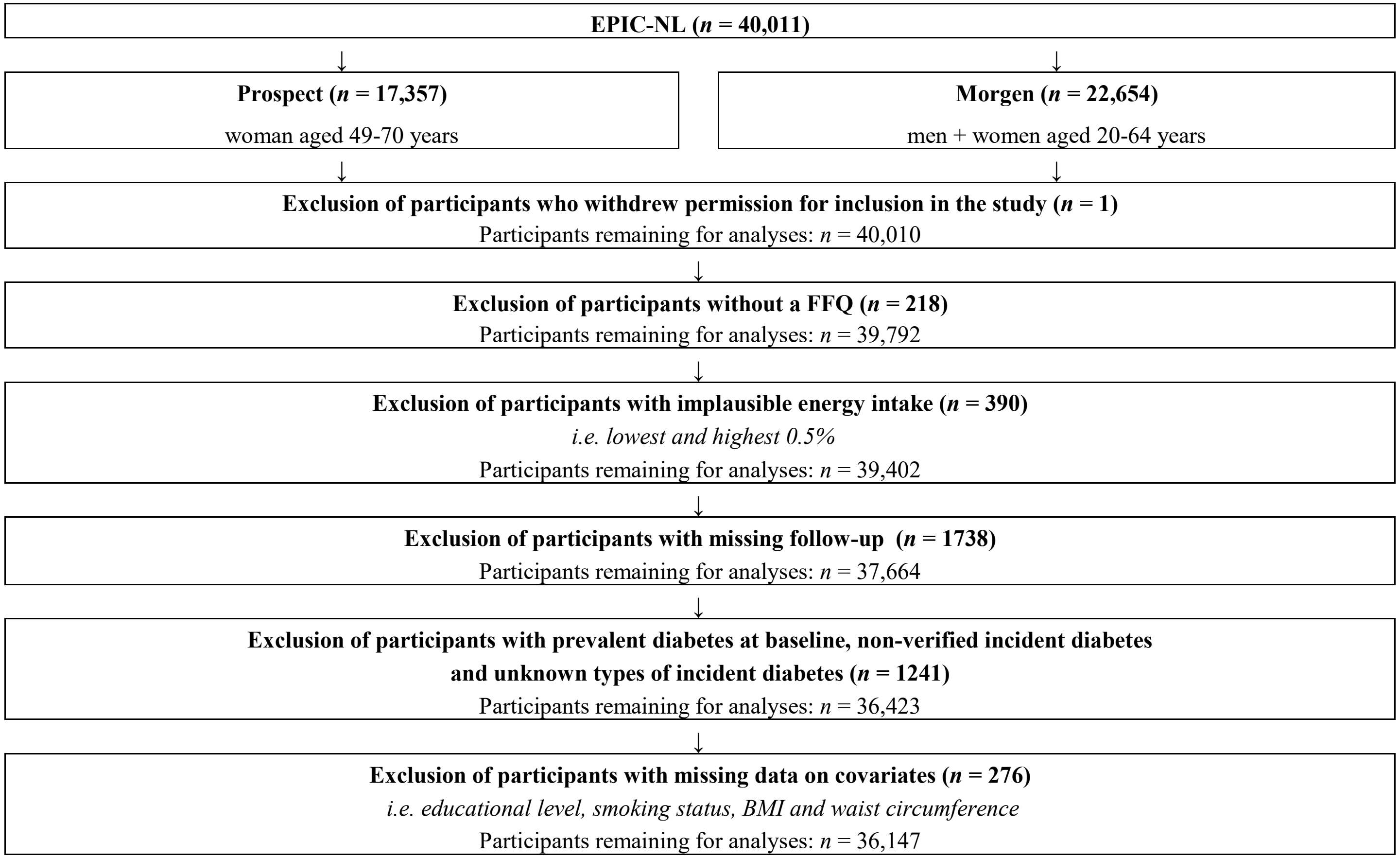

Supplemental figure 1. Flow-chart of participants excluded from the study
